# Public-health risk expression and governance of low-threshold exercise and health content on Douyin short-video platforms: a case study of follow-along fitness short videos

**DOI:** 10.3389/fpubh.2026.1985289

**Published:** 2026-10-20

**Authors:** Yigang Zhang, Zhi Qu, Xiaoyan Guo, Yilin Zhao

**Affiliations:** Law School, Qufu Normal University, Rizhao, China

**Keywords:** exercise and health communication, expressive boundary, low-threshold follow-along fitness short videos, platform governance, risk production

## Abstract

**Introduction:**

Follow-along exercise and health short videos provide the public with convenient exercise references, yet their efficacy statements, applicability conditions, and risk information warrant attention.

**Methods:**

Using a sample of 250 videos from Douyin, this study employed a diagnostic evaluative content analysis to examine efficacy claims, applicability boundaries, risk warnings, and professional justification. Intercoder reliability was assessed, and comparisons were conducted across content types.

**Results:**

The proportions of videos meeting the severe expressive-problem criterion (score 3) were 36.8% for risk warnings, 34.4% for efficacy claims, and 25.6% for applicability boundaries, compared with 2.0% for professional justification. A total of 54.4% of the videos exhibited at least one score-3 problem, and 36.0% exhibited two or more. Content type was statistically associated with score-3 distributions for Dimensions B, C, and D (all Holm-adjusted *p* < 0.001), whereas Dimension E did not reach significance (*p* = 0.369). Fat-loss and body-shaping videos were more likely to show efficacy-claim problems, while pain-relief and general rehabilitation videos were more likely to show problems with applicability boundaries and risk warnings.

**Discussion:**

Governance of follow-along content should be tailored to specific contexts, improving efficacy statements, applicability conditions, risk warnings, and the articulation of professional justification.

## 1 Introduction

Driven by the “Healthy China” strategy and nationwide fitness policies, exercise has been integrated into a policy framework that connects health promotion, disease prevention, and rehabilitation services (1, 2). These policies explicitly call for the development of a scientific fitness system, the implementation of an integrated approach to sports and medicine, and the reinforcement of risk prevention and safety standards. The Physical Activity Guidelines for Chinese (2021) provide age- and health-status-based recommendations, indicating that exercise and health guidance should be tailored to individual conditions with clear applicability requirements and precautions (3). Taken together, these policies suggest that exercise and health content for the general public should not merely demonstrate movements, but also specify target populations, exercise intensity, and necessary precautions.

When such content is disseminated via short-video platforms, whether its expression aligns with the above policy requirements becomes a question worth examining. Short-video platforms have already become a significant arena where the public accesses, searches for, and initially understands health information. Research indicates that the easy accessibility, visual salience, and strong interactivity of short-video platforms are transforming how individuals obtain and search for health information (4, 5). In China, as of June 2025, the number of short-video users had reached 1.068 billion, demonstrating the extensive information reach of these platforms (6). When exercise and health information is presented in the form of movement demonstrations and follow-along routines, the distance between viewing and performing is further compressed, and video content thus acquires the potential to shift from “viewing” to “doing”.

This executability means that follow-along content carries both informational and bodily-practice orientations. Previous research has used the concept of “participability” to explain how imitation and following along translate into bodily practice (7, 8), and user surveys have found associations between information quality, source credibility, and willingness to engage in exercise (9). Research on exercise participation thus raises a further question: do videos also provide the information users need to select movements and judge applicability conditions? Studies on scoliosis-related videos have found that their reliability, information quality, and educational suitability remain inadequate (10, 11).

Accordingly, this study defines low-threshold follow-along exercise and health short videos on the Douyin platform as publicly available videos aimed at ordinary users, oriented toward physical improvement, and directly imitable or followable, and confines the notion of “risk production” to deficiencies in risk information at the expressive level. It examines the expressive conditions and problematic emphases of different content types along four dimensions: efficacy claims, applicability boundaries, risk warnings, and professional justification. Our analysis focuses on observable and verifiable textual, audio, movement demonstration, and account information in publicly available videos, without evaluating the clinical efficacy of individual movements, tracing actual user injuries, or directly equating expressive problems within the sample with platform governance outcomes. Its public health governance value lies in translating scientific fitness and risk prevention requirements into observable content evaluation items (2), thereby providing empirical evidence for risk-warning design and expressive standards in follow-along content.

## 2 Literature review

### 2.1 Defining Core concepts

Studies on short videos about exercise, fitness, and health have not adopted fully consistent terminology. Some emphasize “fitness short videos”, others use “sports short videos”, and still others situate the discussion within short-video health communication or health science popularization (12, 13). The overlapping core of these concepts refers to video content disseminated via mobile short-video platforms, oriented toward ordinary users, featuring movement demonstrations, exercise techniques, or daily fitness sharing, and carrying implications for physical activity, body management, or health improvement (14).

“Low-threshold follow-along fitness short videos” are better understood as a content format characterized by both low viewing thresholds and low following-along thresholds. Rather than belonging to professional training settings, they enter the everyday exercise information context in which ordinary users can quickly access, understand, and attempt to imitate movements (15, 16). The notion of “low threshold” operates on two levels. At the media level, short videos adapt to fragmented time and mobile scenarios, and reduce the barriers to accessing health information through images, subtitles, voiceovers, and movement visuals (17–19). At the bodily execution level, once movement content is presented in an imitable, duettable, or followable manner, users are likely to translate viewing into bodily practice (20). Low threshold, however, does not imply low risk (21). Physical activity guidelines and health promotion policies emphasize that populations of different ages, physical conditions, and health needs vary in appropriate exercise modes, intensities, and precautions (3). Accordingly, this study defines low-threshold follow-along fitness short videos as fitness-oriented video content directed at ordinary users, disseminated via short-video platforms, centered on imitable or followable movement demonstrations, and oriented toward health improvement, body management, or physical activity participation.

### 2.2 Risk society and mediatized risk

The German sociologist Ulrich Beck, in Risk Society, argues that modern industrial society has entered a stage of risk society—while science and technology drive social progress, they also generate large-scale “manufactured uncertainties”, which manifest as risks and become the defining feature of modern society. Unlike natural risks, modern risks are characterized by their manufactured nature and their dependence on knowledge; their sources lie not in external natural forces but in the operational logic of modernity itself (22). Beck also introduced the concept of “organized irresponsibility”, noting that risk producers protect their own interests at the expense of risk bearers (23).

Anthony Giddens distinguishes between “external risks” and “manufactured risks”, arguing that the latter marks the transition from industrial society to risk society. External risks originate mainly from natural forces, whereas manufactured risks are products of modernity itself—consequences of the operation of human knowledge systems, technological systems, and institutional arrangements. Giddens also proposes the concept of “expert systems”, referring to the professional knowledge systems and institutional networks on which people rely to guard against risks—yet these systems themselves may generate new risks through technical failures, miscalculations, or cognitive conflicts among different expert systems (24). In Modernity and Self-Identity, Giddens further observes that “manufactured uncertainty” destabilizes the trust structures of modern society—when the expert systems on which individuals rely for risk assessment become unreliable, people fall into trust anxiety, triggering a crisis of “disembedding” in individual existence (25).

Beck and Giddens together provide a theoretical framework for analyzing risk in low-threshold follow-along fitness short videos (26–28). Short-video health content can be seen as a typical case of “manufactured risk”—its risks are not naturally occurring, but co-constructed by information production, algorithmic distribution, and user following-along behaviors. The “knowledge dependence” of modern risks means that their identification and assessment depend heavily on expert knowledge. When short videos lack professional grounding or risk warnings, ordinary users lack the cognitive reference points needed to assess risk. Risk society theory also reveals the process of “individualization, “in which individuals are detached from traditional support systems and forced to make risk decisions alone. Users of low-threshold follow-along fitness short videos are precisely in this situation: without professional guidance, they must judge for themselves “whether this video suits me” and “whether this movement is safe” (22, 25). Existing research has applied risk society theory to short-video communication analysis, showing that short videos are “both innovators of communication and amplifiers and producers of risk” (29), and that they “interweave and connect risks across the real and virtual worlds” (30). Whether a video provides adequate applicability boundaries and risk warnings directly determines whether such individual risk judgments can be made effectively.

### 2.3 Short-video health communication contexts

Short videos have become a significant digital arena in which the public accesses and searches for health information. Their visual nature, easy accessibility, fast pace, and strong interactivity make health information more readily available in everyday information encounters (31, 32). According to the 56th Statistical Report on Internet Development in China from the China Internet Network Information Center, by June 2025, China had 1.068 billion short-video users and 1.085 billion online video users, indicating the broad social reach of short-video and online video platforms (6).

Domestic health communication research has noted changes in the supply of health information on short-video platforms at an early stage. These platforms host institutional accounts, media accounts, medical professional accounts, and individual science popularization accounts; health communication no longer relies solely on one-way transmission by traditional media (33, 34). Studies of official media accounts and local health science accounts show that news media, public health institutions, and professional knowledge accounts have all entered the short-video space, making source visibility, identity markers, and accountability more salient in health content (35, 36). Research on physician-verified accounts also suggests that professional identity is an important trust cue in short-video health content, but that account authorship, technical packaging, posting time, and presentation format all affect communication outcomes—professional identity does not automatically translate into high-quality communication (37).

Platform recommendation mechanisms have profoundly affected the visibility of health information, but “being recommended” does not equal “being effectively received” (38). Research on science popularization short videos further shows that platformization is reshaping how knowledge is expressed and disseminated (39, 40)—when health content is presented in a follow-along format, changes in the communication context affect not only “what is seen”, but also what ordinary users are likely to “do”.

### 2.4 Health information quality and risk expression

Shifting the focus from communication context to content quality, research on health-information short videos repeatedly cautions that view counts, likes, and shares cannot substitute for judgments about information reliability, completeness, and educational suitability (41, 42). Health information quality assessment commonly uses tools such as DISCERN, JAMA benchmarks, and the Global Quality Scale, focusing, respectively, on information reliability, source transparency, overall quality, and content usability. Evaluations of exercise instruction videos further attend to exercise cycles, target areas, expected effects, precautions, and movement rationales, because these directly affect whether users can judge whether they are suited to practice, how to practice, and when to stop (43).

Empirical research on specific exercise-health topics shows that health and exercise content on short-video and video platforms frequently suffers from insufficient reliability or incomplete explanations (44, 45). A study on lumbar disc herniation-related short videos found that samples across multiple Chinese platforms scored low in overall quality, reliability, and content completeness, with variations across platforms and uploader types (46). For patients with lumbar disc herniation, incomplete content means they may be unable to determine whether a given movement applies to their condition, increasing the risk of inappropriate following-along. Research on knee osteoarthritis-related Douyin videos similarly found low DISCERN, JAMA, and GQS scores, indicating that exercise-health content with public health relevance on these platforms does not necessarily provide adequate information quality (42). Knee osteoarthritis patients are mostly middle-aged and older adults, with specific exercise contraindications and intensity limitations; if videos fail to specify applicable conditions and risk warnings, users may practice inappropriately without professional assessment. Studies on low-back-pain therapeutic exercise videos show that videos uploaded by clinical personnel score higher in reliability and overall quality than those by non-medical users, suggesting that uploaders’ professional backgrounds affect the quality of exercise instruction information (44). Non-professionals’ low-back-pain videos carry higher risk—users may unknowingly imitate movements unsuitable for their spinal conditions. Research on geriatric exercise videos finds that overall quality ranges from moderate to poor, emphasizing the need for more reliable, higher-quality exercise guidance (45). Older adults often have multiple underlying conditions, with narrower safety margins for exercise intensity and movement amplitude; low-quality videos are more likely to expose this population to unnecessary risk. Research on sports nutrition TikTok videos shows, from an adjacent topic, that sports-health information on short-video platforms may also suffer from insufficient educational quality and source reliability (47). Though sports nutrition information does not directly involve movement execution, its misleading advice can still affect users’ exercise performance and recovery.

Collectively, these studies cover different diseases and populations, but converge on a common judgment: when video content addresses users with existing symptoms or special physical conditions, missing information may directly affect users’ assessment of their own suitability and increase the risk of inappropriate following-along.

These studies provide evaluative reference points for this study, but they focus mostly on specific diseases, rehabilitation training, geriatric exercise, or sports nutrition, and cannot substitute for analysis of general low-threshold follow-along fitness short videos. For content aimed at ordinary users that can be directly imitated and followed, the evaluative emphasis needs to shift from general information quality to specific questions: whether efficacy claims are excessive, whether target populations are specified, whether risk warnings are adequate, and whether movement rationales are comprehensible.

### 2.5 Platform governance and expressive boundaries

Platform governance research has moved the discussion of health short videos from the narrow question of whether content is true or false to structural dimensions such as source visibility, information disclosure, and recommendation mechanisms (48–50). Research on information disclosure in science communication shows that content producer identity, commercial relationships, and backstage information affect audience trust in communicators and their content (48). Studies on health science popularization short videos also indicate that account verification, professional identity, and content packaging affect public understanding and acceptance of health information, confirming that identity markers and source attribution are important components of health communication (51).

Research on health misinformation reminds us that risk does not come only from entirely false health claims, but also from one-sided interpretations, faulty generalizations, and misleading behavioral recommendations. This is precisely the “gray-zone problem” in low-threshold follow-along fitness short videos: content may not be entirely wrong, but excessive efficacy claims, unclear target populations, insufficient risk warnings, or vague professional grounding can nevertheless undermine ordinary users’ capacity for basic suitability and risk assessment (52). This observation is precisely the starting point for this study’s use of “expressive boundary” as an analytical framework. Research has also examined electronic health literacy content on TikTok (53).

### 2.6 Literature review and research positioning

Existing literature provides three foundations for this study: research on short-video health communication has shown how platformization, visualization, multi-actor participation, and recommendation mechanisms reshape health information contact; research on fitness short videos has shown how participability and following-along behaviors translate viewing into bodily practice; and research on health information quality has provided evaluative references for reliability, completeness, educational suitability, and source transparency. What these bodies of work have not yet fully connected is the question of how low-threshold follow-along fitness short videos—aimed at ordinary users—can present efficacy claims, applicability conditions, risk warnings, and professional grounding simultaneously.

This study takes low-threshold follow-along fitness short videos as an independent object of analysis, examining how different content types express efficacy claims, applicability conditions, risk warnings, and professional grounding. We do not test whether videos directly cause user harm, nor do we evaluate creators’ actual professional competence. Rather, it diagnoses whether the expressive information in publicly available videos is sufficient to support ordinary users in forming basic suitability and risk judgments. This positioning links research on short-video health communication, fitness short-video participation studies, health information quality assessment, and platform governance discussions, providing a literature foundation for analyzing expressive problems across different video types and their governance implications.

## 3 Methods

### 3.1 Research design and sample

This study uses a diagnostic evaluative content-analysis method, with the specific workflow illustrated in Figure 1. Our analysis focuses on whether low-threshold follow-along exercise-health short videos exhibit problems at the expressive level, including excessive efficacy claims, unclear applicability boundaries, inadequate risk warnings, or vague professional justifications. This study positions itself as “content diagnosis” rather than “causal testing”—that is, it does not evaluate the clinical efficacy of specific movements, nor does it trace users’ physical responses after following along. The sample is limited to publicly visible videos on Douyin, primarily because the platform has a relatively high concentration of low-threshold follow-along content with considerable variety. Additionally, the title, cover, caption, subtitles, voiceover, and visuals of each video are directly observable and repeatedly verifiable, making systematic coding and cross-type comparison feasible.

**Figure 1:**
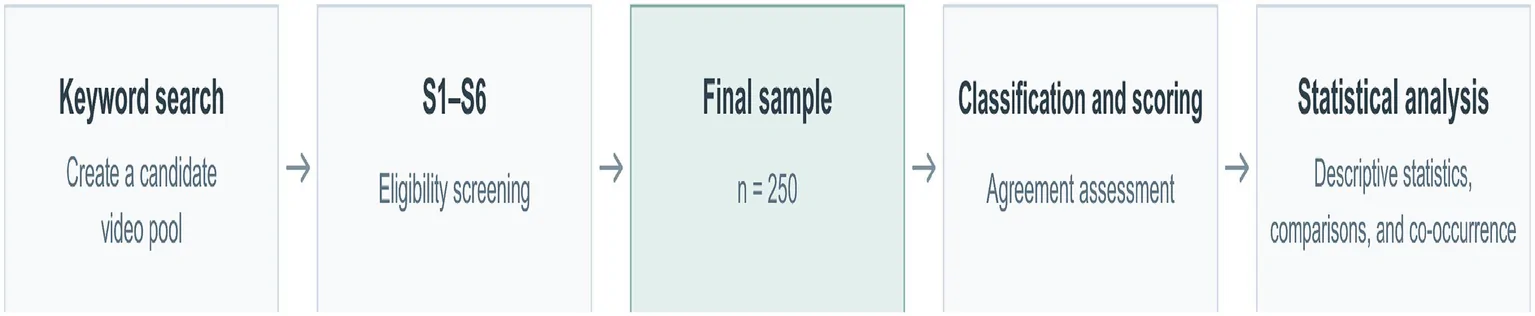
Research workflow.

Sample inclusion followed all six criteria S1–S6: public verifiability, orientation toward ordinary users, a low-threshold follow-along format, a health or physical-improvement orientation, a non-medical diagnostic or prescriptive focus, and a non-commercial primary purpose; videos that did not meet these criteria were excluded. A total of 250 videos were selected, with 50 from each of the five target strata (Table 1). The video coverage period spanned from January 2024 to July 2026; the collection dates recorded in the formal sample sheet were 17–19 August 2026. Retrieval followed the platform’s default ranking order, and videos were screened one by one using criteria S1–S6. Sampling continued within each stratum until 50 videos were collected, and duplicate videos were retained only once. After collection, the samples were reclassified and recoded according to the coding scheme. Details of the research subjects and sample scope are presented in Table 2. The five strata were used for sampling quotas; the A content categories were reassigned during formal coding according to each video’s primary goal. The platform’s default ranking affects which videos enter the quota sample, which is not a probability sample.

**Table 1:** Sampling target strata and sample configuration.

Sampling target strata	Representative search topics/keywords	Target sample size	Percentage
Posture correction	Rounded shoulders, dowager’s hump, anterior pelvic tilt, posture correction, follow along	50	20.0%
Pain relief/general rehabilitation expression	Low back pain, neck and shoulder pain, knee pain, sciatica, rehabilitation exercises	50	20.0%
Fat loss and body shaping	Full-body slim, belly fat loss, arm slimming, fat burning, sweat session, body shaping	50	20.0%
Stretching/desk exercises/fragmented movement	Bedtime stretching, desk stretching, office stretching, desk exercises, 5-min stretch	50	20.0%
Baduanjin/wellness exercises	Baduanjin, wellness exercises, traditional fitness routines, meridian exercises, health qigong	50	20.0%
Total	Not applicable	250	100.0%

**Table 2:** Research subjects and sample scope description.

Category	Content	Description
Research subjects	Low-threshold follow-along exercise-health short videos on Douyin	Focus on videos that ordinary users can directly follow along with and that have health-improvement orientations
Unit of observation	Individual publicly available videos	Each single video serves as the smallest analytical unit
Data source	Publicly searchable videos on Douyin	Only videos that are queryable, viewable, and verifiable are included
Sample scope	Stretching, posture correction, pain relief/general rehabilitation, fat loss and body shaping, Baduanjin/wellness exercises, desk exercises, etc.	Low-threshold follow-along content consistent with the research framework
Inclusion criteria	Publicly verifiable; oriented toward ordinary users; directly followable; health-improvement oriented; non-medical diagnostic/prescriptive; non-commercially driven	Exclusion if any criterion is not met
Exclusion criteria	Broken links, live-stream replays, medical prescriptions, commercially driven content, unverifiable content, duplicate samples, inconsistent with the sampling frame	Ensures sample consistency and comparability
Sample size	250 videos	Configured as 50 per sampling stratum; final A content types were reassigned during coding.
Classification approach	A dimension for content type; B–E dimensions for boundary expression scoring	A for classification; B–E for evaluating boundary expression
Coding basis	Titles, cover text, captions, voiceover subtitles, movement demonstrations, and account information	Primary basis for formal coding
Video coverage period	January 2024–July 2026	Period covered by the sampled video content.
Collection dates	17–19 August 2026	Dates recorded in the formal sample sheet.

### 3.2 Sample screening and coding framework

#### 3.2.1 S1–S6 sample screening criteria

To determine whether candidate videos fall within the scope of the research subjects, this study established the S1–S6 screening criteria to assess video verifiability, orientation toward ordinary users, low-threshold follow-along characteristics, health/body improvement orientation, non-medical prescription features, and non-commercial dominance. The specific judgment rules are presented in Table 3. Candidate videos must simultaneously meet all six criteria; if any criterion is judged as “no” or coded as “0”, the video is excluded from the formal sample and does not proceed to subsequent coding in the A content type or B–E evaluation dimensions. S1–S6 are used solely for screening research materials, not for judging whether videos exhibit boundary expression problems. Samples that pass the screening are then classified and evaluated according to the formal coding rules.

**Table 3:** S1–S6 screening criteria and judgment rules.

No.	Screening criterion	Judgment points
S1	Publicly available and verifiable	The video link is accessible; content can be reviewed; title, cover, subtitles, and visual information are verifiable.
S2	Oriented toward ordinary users	The video targets the general public, rather than professional athletes, coaches, medical practitioners, or specific training groups.
S3	Low-threshold and followable	Movement steps are clear, rhythm is followable, and ordinary users can imitate without professional guidance.
S4	Health/body improvement oriented	Content revolves around health promotion, postural improvement, pain relief, relaxation and recovery, or body management.
S5	Non-medical diagnosis/prescription	Does not focus on disease diagnosis, treatment prescriptions, clinical rehabilitation plans, or medical advice.
S6	Non-commercially driven	Content is primarily health knowledge or follow-along guidance; commercial promotion is not the core purpose.

#### 3.2.2 A content type classification

Dimension A is used to identify the primary content type of each video. It serves as a content classification and is not employed for risk scoring. During coding, the title, primary goal, and main efficacy claim are used as the basis for primary classification; if a video involves multiple themes, it is assigned to one main category according to its most prominent content orientation. The operational definitions of A1–A5 are presented in Table 4. A classification identifies a video’s primary goal, whereas B–E scoring examines its mode of expression; the two draw on overlapping materials. For example, “slimming” may serve as a goal cue for A3, while B scoring further determines whether the video contains rapid-results or strong-guarantee expressions. Pain-related content involves a specific physical condition, and C and D scoring accordingly check for applicable restrictions and stopping conditions. Type comparisons are therefore shaped by both content characteristics and scoring rules. This distinction clarifies the respective judgment objects and coding sequence of A content types and B–E evaluation dimensions, but it does not imply that content goals and expressive problems are conceptually unrelated.

**Table 4:** Operational definitions of a content types.

Category	Operational definition	Primary identification criteria	Classification rule
A1: Posture correction	Follow-along videos primarily targeting postural issues such as rounded shoulders, kyphosis, anterior pelvic tilt, forward head posture, and uneven shoulders.	Titles, covers, or voiceovers contain expressions such as “posture correction”, “rounded shoulder improvement”, “kyphosis correction”, or “anterior pelvic tilt adjustment”; movements typically center on postural realignment, spinal alignment, and opening of the shoulders and neck.	Exclude general stretching or relaxation without a stated postural goal.
A2: Pain relief/general rehabilitation	Follow-along videos primarily targeting the relief of neck, shoulder, low back, knee, muscular tension, or general physical discomfort.	Expressions such as “pain relief”, “neck and shoulder relaxation”, “low back pain improvement”, “general rehabilitation”, or “discomfort relief” appear; movements emphasize soothing, releasing, activating, or functional restoration.	Exclude disease treatment, prescribed exercise, or a clinical rehabilitation program under S5.
A3: Fat loss and body shaping	Follow-along videos primarily targeting fat loss, weight reduction, body shaping, and improvement of body contours.	Expressions such as “fat loss”, “calorie burning”, “belly fat loss”, “leg slimming”, “body shaping”, or “six-pack abs training” appear; movement structures typically emphasize calorie expenditure, full-body engagement, or localized sculpting.	General aerobic activity without an explicit fat-loss or body-shaping goal is not prioritized for this category.
A4: Stretching/desk exercises/fragmented movement	Follow-along videos primarily targeting stretching, relaxation, relief from prolonged sitting, and office break activities.	Expressions such as “stretching”, “relaxation”, “desk exercises”, “must-do for desk workers”, “office workers”, or “office stretching” appear; movements are typically low in intensity and slower in pace, suitable for fragmented scenarios.	Exclude content primarily focused on posture correction, pain relief, or specialized training.
A5: Baduanjin/wellness exercises	Follow-along videos primarily featuring Baduanjin, traditional wellness exercises, and Daoyin breathing and regulating exercises.	Titles, covers, or voiceovers explicitly mention “Baduanjin”, “wellness exercises”, “Daoyin”, “breath regulation”, or “traditional wellness”; movements exhibit clear characteristics of traditional Chinese exercise regimens.	Exclude general fitness routines that merely use traditional imagery or terminology.

The classification basis for Dimension A is the video’s “goal orientation” (i.e., which type of physical improvement the video targets), rather than the “intensity of claims” or “mode of expression” through which that goal is articulated in the video—the latter two are assessed separately by Dimension B (efficacy claims) and Dimension D (risk warnings).

#### 3.2.3 B–E four evaluation dimensions

Dimensions B–E are used to assess specific aspects of boundary expression in the videos. Dimension B examines whether efficacy claims are clear and proportionate; Dimension C examines whether target populations and usage conditions are clearly specified; Dimension D examines whether risk warnings are adequate; and Dimension E examines whether movement rationales, professional sources, and explanatory transparency are provided. The four dimensions are coded according to the operational definitions in Table 5 and the dimension-specific rating standards in Table 6.

**Table 5:** Operational definitions of B–E evaluation dimensions.

Dimension	Operational definition	Key indicators
B: Efficacy claims	Determines whether the video makes explicit claims, implications, or reinforcements about exercise effects, and establishes a strong causal link between movements and outcomes.	Whether determinative or absolutist expressions such as “quick results”, “immediate improvement”, “one routine works”, or “lose weight in days” appear.
C: Applicability boundaries	Determines whether the video specifies who is suitable to practice, who is not, and under what conditions one may practice.	Whether applicable populations, physical conditions, age ranges, contraindications, and prerequisites for practice are stated.
D: Risk warnings	Determines whether the video provides warnings about potential physical risks, stopping conditions, or precautions during follow-along practice.	Whether movement intensity, pain signals, risk areas, contraindicated movements, and stopping conditions are specified.
E: Professional justification	Determines whether the video provides sources or rationales for the movements, methods, or recommendations, enabling traceable support for the content.	Whether creator identity, professional background, course sources, teaching materials, traditional practice sources, or research bases are stated.

**Table 6:** Dimension-specific scoring criteria and evaluation questions.

Dimension	Evaluation question	Score 1	Score 2	Score 3	Key exceptions
B: Efficacy claims	Does the video establish an excessively strong link between a movement and a physical or health outcome?	Movement demonstration or ordinary activity without a definite promise of a physical outcome.	General improvement, relief, body-shaping, or wellness claims without rapid, absolute, or therapeutic promises.	Rapid-result claims, strong guarantees, absolute or therapeutic claims, or a strong link between an easy movement and a substantial outcome.	“One move”, “one week”, or “intense sweating” alone does not trigger 3; the outcome claim must be examined.
C: Applicability boundaries	Does the video specify suitable and unsuitable users and practice conditions?	Suitable users and context-appropriate restrictions, alternatives, or stopping conditions are specified.	Audience cues or practice conditions are general or incomplete.	Explicit or implicit suitability for everyone or all physical conditions, or generalized follow-along advice for people with pain or rehabilitation needs.	“Beginner” or “no experience needed” does not automatically trigger 3; generalized suitability also applies to low-intensity content.
D: Risk warnings	Does the video explain risks, contraindications, stopping conditions, or necessary precautions?	Specific warnings, contraindications, stopping conditions, or advice to seek care are appropriate to the context.	Warnings are vague or inconspicuous, or are absent from ordinary low-intensity content.	Necessary warnings are missing in pain, rehabilitation, high-intensity, loaded, highly repetitive, or high-impact contexts; or pain is minimized or risky practice encouraged.	*B* = 3 does not imply *D* = 3. High intensity, loading, or impact requires observable evidence.
E: Professional justification	Does the video state professional identity, movement sources, or methodological justification?	Ordinary low-intensity sharing without strong health claims, or identity, sources, or movement rationale proportionate to the claim.	Identity or rationale cues are weak, or a health claim is made without explaining its source or supporting basis.	Unverifiable authority cues accompany strong health or therapeutic claims, or the claimed professional identity clearly does not match the therapeutic claim.	A self-described coach or an account name is neither sufficient justification nor an automatic trigger for 3.

Score 0 denotes insufficient material; no score-0 observations occur in the reported sample. Scores 1–3 indicate no evident problem, general insufficiency, and the dimension-specific severe-problem criterion. Each score of 3 requires a recorded trigger.

#### 3.2.4 Scoring rules and exception cases

Scoring levels are defined by the frozen coding manual. A score of 0 indicates only insufficient material and is not included in severity ranking; scores of 1–3 denote, respectively, no evident problem, general insufficiency, and meeting the criterion for a severe problem. Each dimension is scored according to its specific trigger conditions. Scientific fitness policies and physical activity guidelines emphasize that exercise guidance should provide corresponding explanations in relation to target populations, practice conditions, and risk contexts. On this basis, this study distinguishes scores of 2 and 3 as dimension-specific operational thresholds, rather than adopting a uniform standard of “whether information is completely absent.” A score of 2 indicates that some expressive cues are present, but the information is vague, incomplete, or inadequately supported. A score of 3 requires the severe trigger conditions specified for that dimension, such as excessively strong efficacy claims, generalized target populations, absence of necessary warnings in specific risk contexts, or unverifiable authoritative packaging combined with strong health claims or therapeutic expressions. A score of 3 therefore indicates that a video meets the preset criterion for a severe expressive problem; it does not mean that actual health harm to users has been demonstrated. The key exception rules are retained separately in Table 7.

**Table 7:** Key exception rules.

Dimension	Key exception rules
B: Efficacy claims	Terms such as “one move”, “one week”, “sweat session”, or “total body slim” do not automatically merit a score of 3. The outcome claim must be examined. General improvement expressions are typically scored as 2; rapid-result promises, strong guarantees, absolute or therapeutic claims, or a strong link between an easy movement and a substantial outcome meet the score-3 criterion.
C: Applicability boundaries	“Beginner” or “no experience needed” does not automatically trigger 3; generalized suitability also applies to low-intensity content. Explicit or implicit suitability for everyone or all physical conditions, or generalized follow-along advice for people with pain or rehabilitation needs.
D: Risk warnings	Low-intensity stretching or general wellness exercises that merely lack general reminders are typically scored as 2. However, pain/general rehabilitation, high-intensity, weight-bearing, high-repetition, or impact content without stopping conditions, contraindications, or risk warnings is usually scored as 3.
E: Professional justification	In the professional justification dimension, a health claim without stated sources, identity, or movement rationale is typically scored as 2. A score of 3 is assigned only when unverifiable authoritative packaging—such as claims to medical, expert, authoritative, or secret-remedy status—is combined with strong health claims or therapeutic expressions, or when professional identity clearly mismatches therapeutic expressions. Account nicknames or vague identity cues do not by themselves constitute sufficient professional justification and do not automatically trigger a score of 3.

### 3.3 Coding process and quality control

Before formal coding, a pilot coding exercise was conducted on 50 videos, with 10 videos drawn from each sampling stratum, in order to identify rule ambiguities and further clarify scoring boundaries. The pilot coding focused on distinguishing general improvement from strong efficacy claims, and object descriptions such as “beginner” or “zero foundation” from generalized applicability. The relevant judgment clarifications were refined into a frozen version of the coding manual.

Coding proceeded through three sequential stages: screening, coding, and review. During screening, each candidate video was checked against S1–S6 one by one; failure to meet any criterion led to exclusion from subsequent stages. Videos that passed screening were first assigned an A content type, then coded on Dimensions B, C, D, and E separately. Coding justifications and key expressions were recorded simultaneously as evidence. After initial coding was completed, a unified review was conducted, focusing on the accuracy of type classification and whether dimensional scores conformed to the judgment rules in Tables 5–7. In cases of uncertainty, the original video was revisited for confirmation. The disagreement review and unresolved items are reported in Table 8.

**Table 8:** Coding disagreements and recorded adjudication.

ID/item	Scores	Result	Evidence summary	Applicable rule	Recorded rationale or unresolved point
21/D	2/3	2	The existing summary states only “involves the lower back.”	*D* = 3 requires a defined risk context, such as pain, rehabilitation, high intensity, loading, or impact, together with missing necessary warnings.	The summary alone does not establish a *D* = 3 trigger. The recorded decision retains *D* = 2 because the evidence does not demonstrate the required risk context and omission.
77/B	3/2	3	Reverse abdominal curls are directly linked to anterior pelvic tilt and postpartum recovery.	*B* = 3 requires rapid results, a strong guarantee, an absolute or therapeutic claim, or a strong link to a substantial outcome.	The recorded rationale treats the link between a single movement and these physical outcomes as strong outcome binding and retains *B* = 3. The full wording should establish why this exceeds a general improvement claim.
81/B	2/3	3	The evidence summary reads “one movement, open the armpits, and gain a good mood.”	*B* = 3 requires an observable strong, absolute, therapeutic, or substantial-outcome claim.	The recorded rationale instead refers to improving rounded shoulders, hunched posture, and forward head posture. These claims must be checked against the same video before the *B* = 3 decision is treated as settled.
90/C	3/1	1 (unresolved)	The review summary mentions the knee; the recorded rationale also refers to rehabilitation exercise.	*C* = 1 requires suitable users and appropriate restrictions, alternatives, or stopping conditions. *C* = 3 requires a generalized suitability trigger.	The source records *C* = 1 but states that it is unsupported without explicit applicability information. If such information is absent, the recorded explanation proposes *C* = 2; if present, the supporting speech, subtitles, or frames must be supplied. The recorded 1 also conflicts with the reported overall C distribution.
153/E	2/1	2	Neck relaxation or improvement is promised without a stated supporting source.	*E* = 2 covers inadequate supporting justification. *E* = 3 additionally requires unverifiable authority plus strong claims, or a clear identity–therapeutic-claim mismatch.	The recorded decision retains *E* = 2 because the improvement claim lacks a specific source or rationale. No *E* = 3 trigger is documented.
238/C	2/3	2	No applicability conditions are identified in the evidence summary.	Missing applicability information alone does not automatically trigger *C* = 3; generalized suitability or the relevant pain/rehabilitation context must be established.	No “suitable for everyone” statement or generalized advice to a pain/rehabilitation population is documented. The recorded decision therefore retains *C* = 2.
244/C	3/2	3	The video states “fitness movements suitable for everyone.”	An explicit claim of suitability for everyone triggers *C* = 3.	The recorded decision is *C* = 3 on the basis of the generalized suitability claim. This reconciles the review score of 2 with the retained rationale stating *C* = 3.

B, C, D, and E denote efficacy claims, applicability boundaries, risk warnings, and professional justification. In the second column, the first and second values are the initial and review scores, respectively; video IDs identify individual sample records. This table presents the recorded disagreement review and does not enter the calculation of coding agreement. Unresolved entries require verification of the original evidence and reconciliation with the analysis dataset.

After the coding manual was frozen, 50 videos were randomly drawn from the 250 formal samples across the five sampling strata, with 10 videos per stratum and a random seed of 20,260,912. Two coders, using the same version of the manual, independently determined the A content type and completed B–E scoring for the same videos, without exchanging results before initial coding was completed, and separately recorded evidence locations and brief excerpts.

Intercoder reliability was calculated using the original scores from before discussion between the two coders. A represented nominal classification and was assessed using unweighted Cohen’s κ; B–E represented ordinal scores and were assessed using linearly weighted κ, with raw agreement rates also reported. Of the 250 judgments, 243 were consistent (97.2%); the κ value for A was 1.000, and those for B–E were 0.945, 0.807, 0.963, and 0.947, respectively. The dimension-specific results are presented in Table 9.

**Table 9:** Coding agreement results.

Coding item	Valid sample size	Complete agreements	Raw agreement rate	Unweighted Cohen’s kappa	Linear-weighted Cohen’s kappa
A: Content category	50	50	100.0%	1.000	Not applicable
B: Efficacy claims	50	48	96.0%	0.934	0.945
C: Applicability boundaries	50	47	94.0%	0.853	0.807
D: Risk warnings	50	49	98.0%	0.961	0.963
E: Professional justification	50	49	98.0%	0.947	0.947

A is nominal and is assessed using unweighted Cohen’s kappa; B–E are ordinal and are assessed using linear-weighted Cohen’s kappa. All statistics use the original scores recorded before discussion, for the same 50 videos.

The two coders produced 2, 3, 1, and 1 disagreements on Dimensions B, C, D, and E, respectively. Table 8 presents the recorded review of these seven disagreements, including pre-discussion scores, evidence summaries, applicable rules, and adjudication rationales. Reliability estimates use the two coders’ original pre-discussion scores, rather than the adjudicated scores. The records for videos 77, 81, and 90 retain unresolved evidence or consistency issues, which require verification against the original videos and reconciliation with the analysis dataset.

### 3.4 Data analysis methods

After coding was completed, frequencies and percentages for the A categories and each B–E dimension were calculated to describe the overall distribution across dimensions. Cross-tabulation analyses were then conducted by A category to compare differences across content types on each dimension. The results of the cross-tabulation were visualized in charts to illustrate the concentration and variation patterns of different video types across dimensions, providing a data foundation for subsequent result analysis. Frequencies and percentages describe content categories and B–E scores. We also summarize the number of score-3 problems per video, pairwise co-occurrence, and mutually exclusive combinations. For type comparisons, each dimension is dichotomized as score 3 versus scores 1–2, producing a 5 × 2 contingency table. Expected counts for B, C, and D meet the requirements for Pearson’s chi-square test; the sparse E table is assessed with a two-sided Fisher–Freeman–Halton exact test. Holm adjustment is applied across the four omnibus tests at a significance level of 0.05, and Cramér’s V is reported as an association measure. These tests assess within-sample associations. Proportions for individual categories are interpreted descriptively rather than as pairwise significance tests.

## 4 Results

### 4.1 Content type composition of the formal sample

From Dimension A, the 250 samples are distributed across five categories of low-threshold follow-along exercise-health content. Posture correction videos are the most numerous, with 60 videos (24.0%), followed by pain relief/general rehabilitation expression videos with 59 (23.6%), fat loss and body shaping videos with 50 (20.0%), Baduanjin/wellness exercise videos with 41 (16.4%), and stretching/desk exercise/fragmented movement videos with 40 (16.0%).

The five categories show relatively balanced sample sizes overall, though Baduanjin/wellness exercises and stretching/desk exercises fall slightly below the original quota target of 50. This is because the five sampling target strata at the sampling stage served only as a reference framework for sample configuration; the A categories are the result of reclassification after coding, as some videos’ actual content orientation did not fully align with their original retrieval categories and were reclassified into other categories after coding. As shown in Figure 2, this distribution provides a foundation for subsequent comparisons.

**Figure 2:**
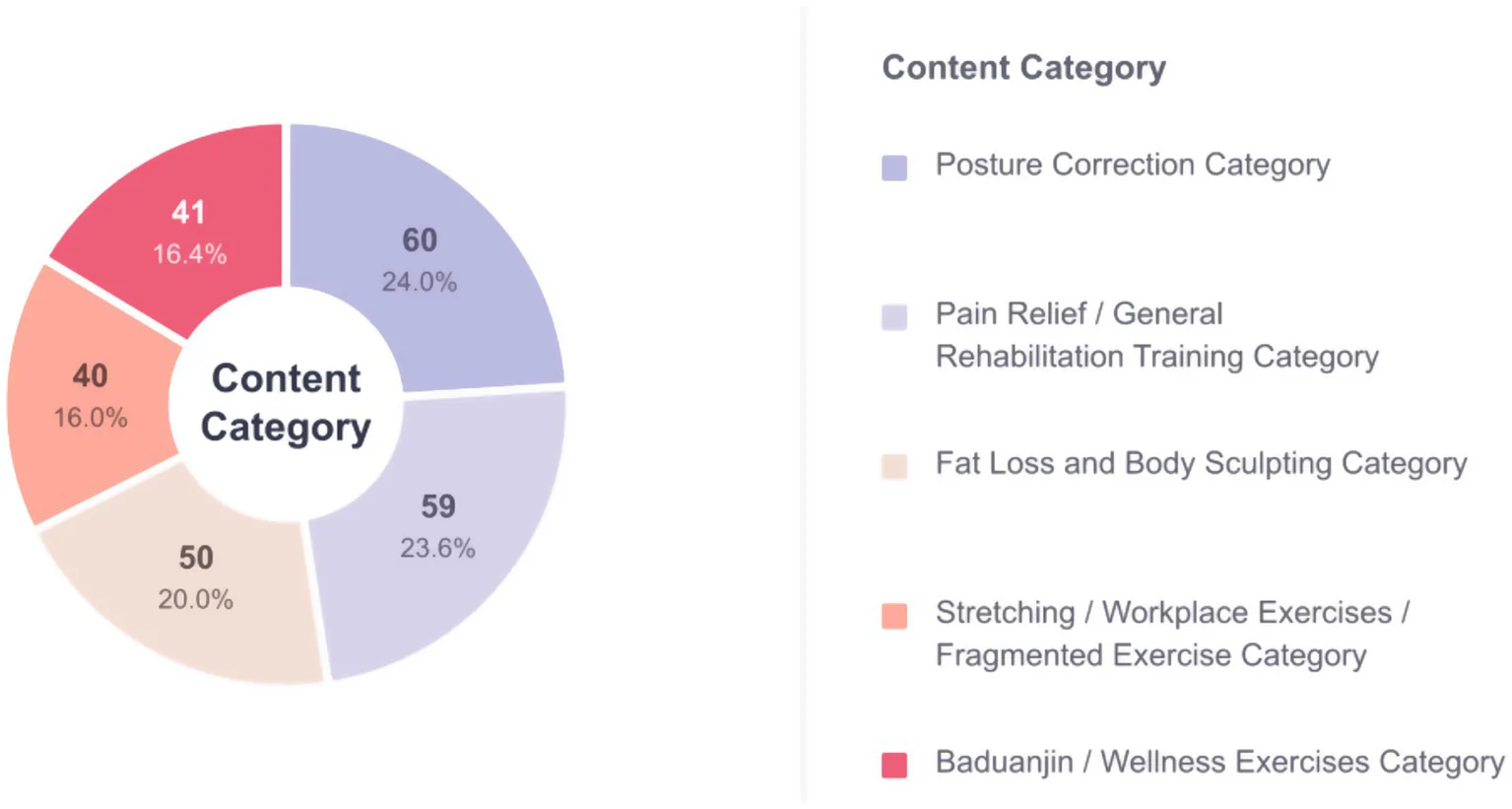
Content type composition chart. *n* = 250, sectors labeled with counts (percentages).

### 4.2 Overall boundary expression status across B–E dimensions

To avoid judging content boundary status based solely on severely problematic samples, this study first presents the full grade distribution across the B–E dimensions. As shown in Figure 3, all four dimensions are dominated by grade 2 and grade 3 samples, with relatively low proportions of grade 1 samples.

**Figure 3:**
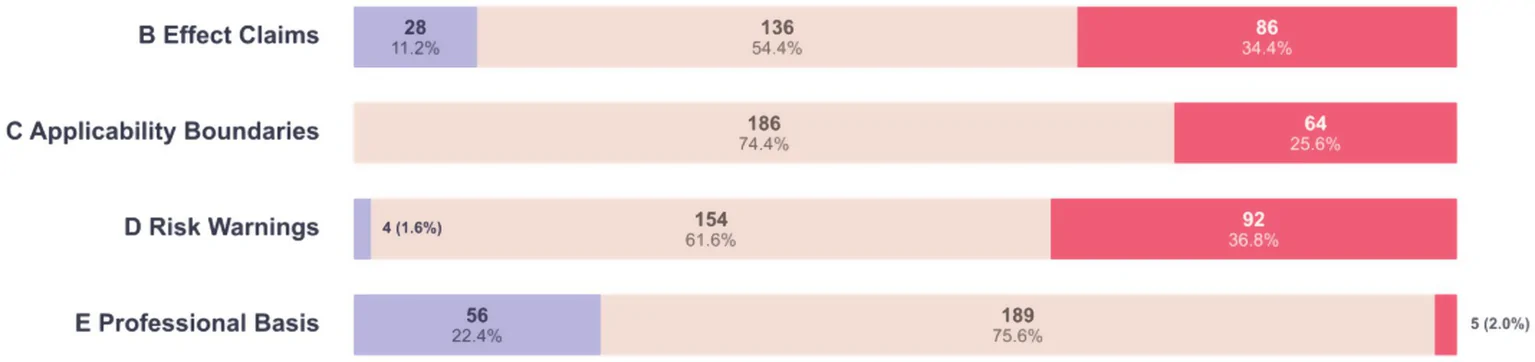
Full grade distribution chart. *n* = 250, labeled with counts (percentages).

In the efficacy claims dimension, 54.4% of samples received grade 2, 34.4% received grade 3, and only 11.2% received grade 1. Relevant expressions primarily used terms such as “improve”, “slim down”, and “relieve discomfort” to directly link movements to physical outcomes. In the applicability boundaries dimension, 74.4% of samples received grade 2, 25.6% received grade 3, and no samples received grade 1. Samples generally failed to simultaneously clarify applicable populations, limitations, or conditions unsuitable for following along. In the risk warnings dimension, 61.6% of samples received grade 2 and 36.8% received grade 3, with grade 3 accounting for the highest proportion among the four dimensions. Insufficient risk warnings were mainly concentrated in the areas of stopping conditions when pain worsens, contraindicated situations, and movement load risks. In the professional justification dimension, 75.6% of samples received grade 2, only 2.0% received grade 3, and 22.4% received grade 1.

In terms of the distribution of severe problems, as shown in Figure 4, insufficient risk warnings were the most frequent, with 92 samples (36.8%), followed by excessive efficacy claims with 86 (34.4%), severely unclear applicability boundaries with 64 (25.6%), and severely vague professional justification with 5 (2.0%). High-frequency problems were mainly concentrated in the three areas of risk warnings, efficacy claims, and applicability boundaries.

**Figure 4:**
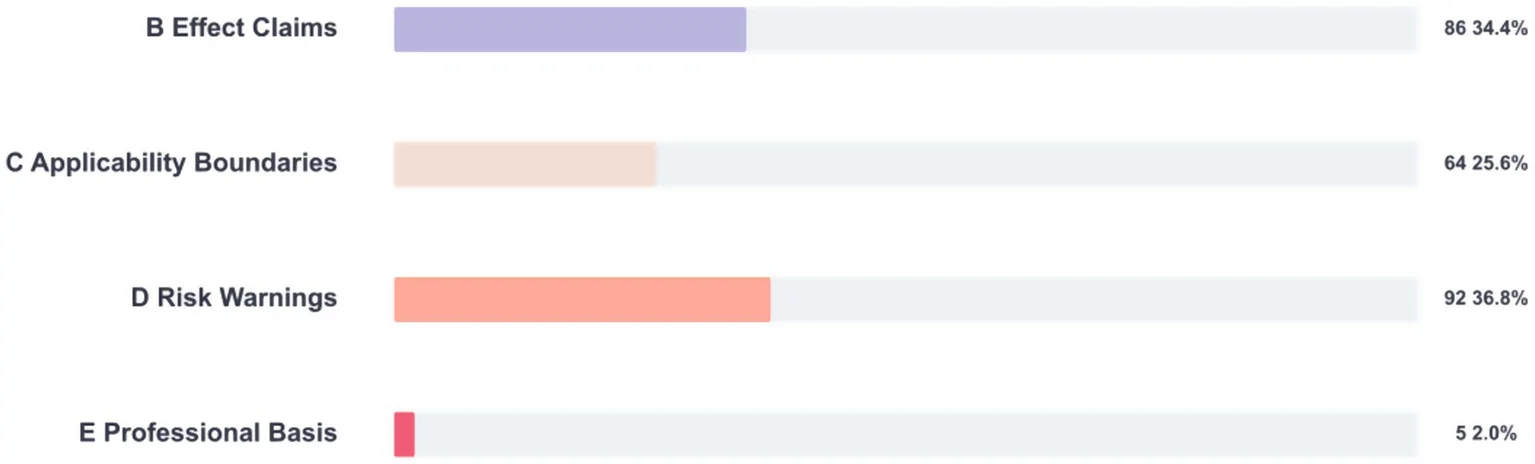
Boundary problem distribution chart. *n* = 250, labeled with percentages.

### 4.3 Differences in boundary expression across content types

The distribution of high-risk boundary expressions varies across content types. Fat loss and body shaping videos show a greater concentration of efficacy claim problems, while pain relief/general rehabilitation expression videos show more prominent problems in applicability boundaries and risk warnings. As shown in Figure 5, the distribution differences across the four dimensions are relatively clear.

**Figure 5:**
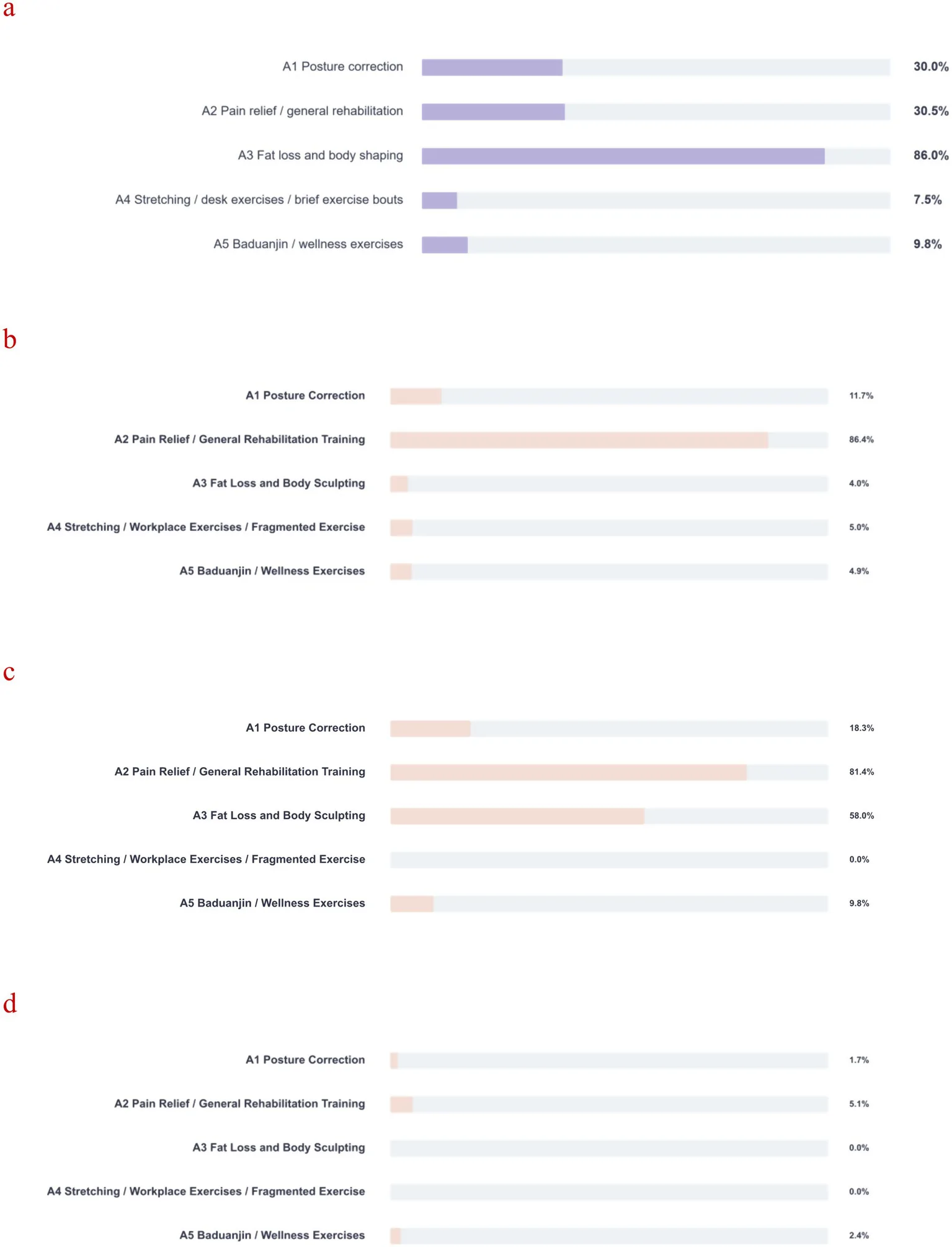
High-risk problem charts for different content types and B–E dimensions. *n* = 250, labeled with percentages, where **(a)** is the efficacy claims problem chart, **(b)** is the applicability boundaries problem chart, **(c)** is the risk warnings problem chart, and **(d)** is the professional justification problem chart.

Looking at the efficacy claims dimension (Figure 5a), fat loss and body shaping videos have the highest proportion of grade 3 at 86.0%, compared to 30.0% for posture correction videos and 30.5% for pain relief/general rehabilitation expression videos. The efficacy claims in these videos mainly revolve around rapid weight loss, localized body shaping, or obvious physical changes. The applicability boundaries dimension presents a different pattern (Figure 5b), where pain relief/general rehabilitation expression videos reach 86.4% in grade 3 proportion, descriptively higher than other categories. This type of content often uses discomfort such as low back pain, neck and shoulder pain, knee pain, or sciatica as entry points, but most videos do not simultaneously clarify unsuitable populations, stopping conditions, or further assessment needs. The risk warnings dimension also shows clear differentiation (Figure 5c). Pain relief/general rehabilitation expression videos have a grade 3 proportion of 81.4%, while fat loss and body shaping videos have 58.0%. The former category’s problems are concentrated in the lack of stopping or medical-seeking guidance in pain and functional discomfort situations, while the latter category shows insufficient risk explanations in high-intensity, continuous movement, weight-bearing, or fat-burning training. In the professional justification dimension (Figure 5d), grade 3 proportions are low across all types, with the highest being only 5.1% for pain relief/general rehabilitation expression videos. The overall exact test for Dimension E did not detect a significant association with content type (*p* = 0.369).

Looking at the composition of high-risk problems within each category (Figure 6), posture correction and fat loss/body shaping videos are mainly composed of efficacy claim problems; pain relief/general rehabilitation expression videos are primarily characterized by applicability boundary and risk warning problems; and stretching/desk exercise/fragmented movement videos have relatively low proportions of high-risk samples, with existing problems still mainly concentrated in efficacy claims and applicability boundaries. Composition proportions use the total number of score-3 problems within each content type; one video may contribute multiple problems.

**Figure 6:**
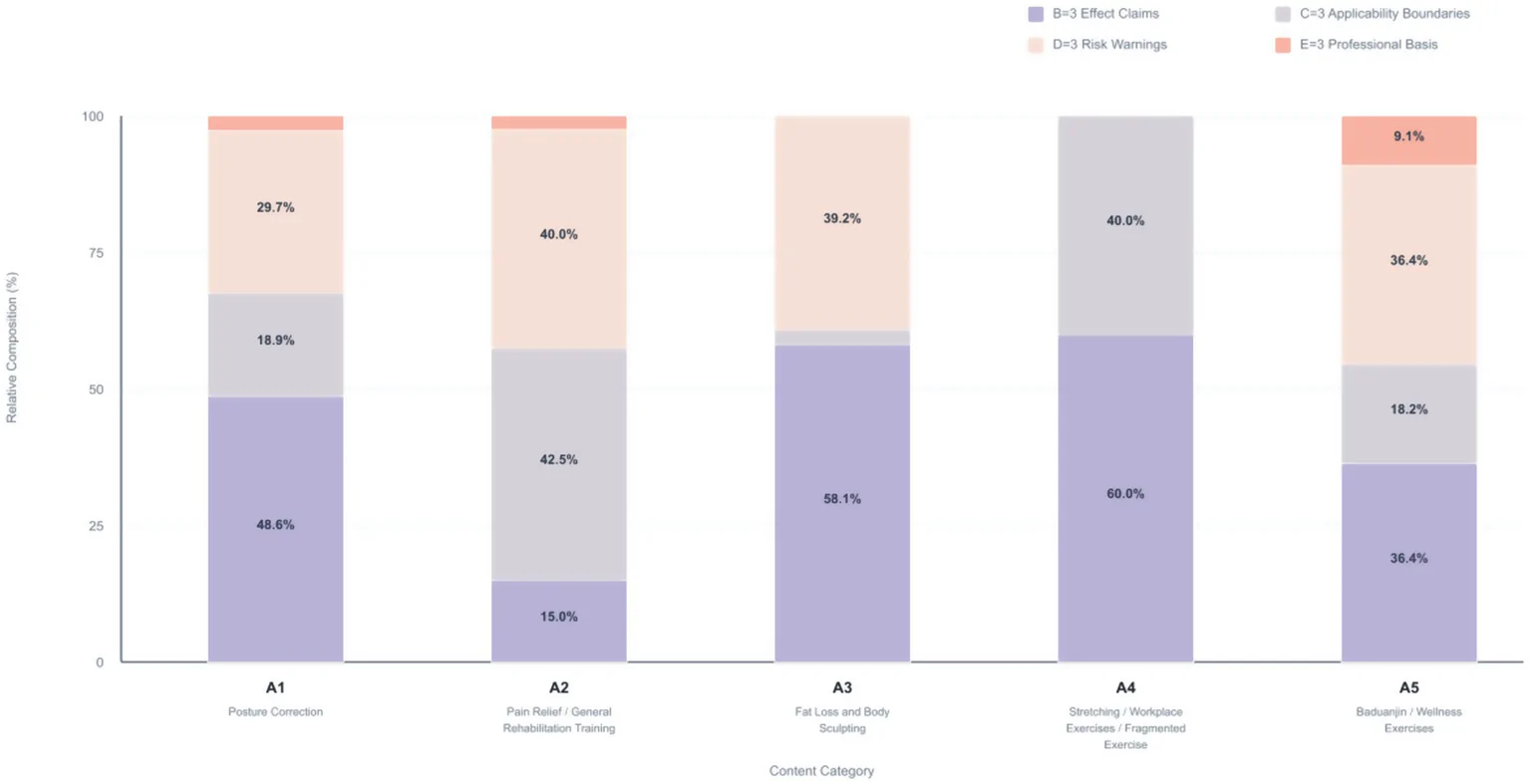
Composition of boundary problems across different content types.

The omnibus association tests are reported in Table 10. Content type was associated with score-3 distributions for Dimensions B, C, and D (all Holm-adjusted *p* < 0.001).

**Table 10:** Score-3 problems by content type and omnibus association tests.

Dimension	A1 (*n* = 60)	A2 (*n* = 59)	A3 (*n* = 50)	A4 (*n* = 40)	A5 (*n* = 41)	Test	*p*	Adjusted *p*	Cramér’s *V*
B: Efficacy claims	18 (30.0%)	18 (30.5%)	43 (86.0%)	3 (7.5%)	4 (9.8%)	Pearson *χ^2^* (4) = 83.765	<0.001	<0.001	0.579
C: Applicability boundaries	7 (11.7%)	51 (86.4%)	2 (4.0%)	2 (5.0%)	2 (4.9%)	Pearson *χ^2^* (4) = 151.183	<0.001	<0.001	0.778
D: Risk warnings	11 (18.3%)	48 (81.4%)	29 (58.0%)	0 (0.0%)	4 (9.8%)	Pearson *χ^2^* (4) = 105.005	<0.001	<0.001	0.648
E: Professional justification	1 (1.7%)	3 (5.1%)	0 (0.0%)	0 (0.0%)	1 (2.4%)	Fisher–Freeman–Halton exact test	0.369	0.369	0.138

Values are counts of score-3 observations and percentages within each category. A1: posture correction; A2: pain relief/general rehabilitation; A3: fat loss and body shaping; A4: stretching/desk exercises/brief exercise bouts; A5: Baduanjin/wellness exercises. B–D use Pearson’s chi-square test; E uses a two-sided Fisher–Freeman–Halton exact test. Holm adjustment covers the four omnibus tests. Cramér’s *V* is calculated from each contingency table. *n* denotes the number of videos in each category; *χ^2^* denotes the Pearson chi-square statistic, with degrees of freedom in parentheses; p denotes the unadjusted probability value; Cramér’s *V* measures association strength.

### 4.4 Accumulation and co-occurrence patterns of high-risk problems

In terms of the number of grade 3 problems per video, 114 samples had no grade 3 problems (45.6%), while 136 samples had at least one grade 3 problem (54.4%). Among these, 90 samples had two or more grade 3 problems simultaneously (36.0%), and 19 samples had three or more grade 3 problems (7.6%). The specific distribution is shown in Figure 7.

**Figure 7:**
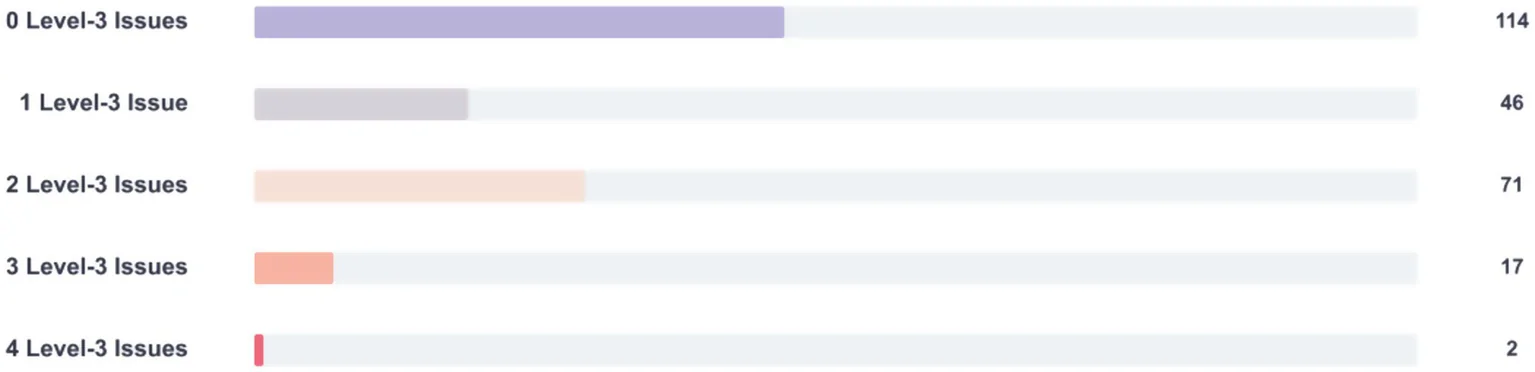
Distribution of high-risk problem counts. *n* = 250, labeled with counts.

In terms of pairwise problem co-occurrence, unclear applicability boundaries and insufficient risk warnings co-occurred most frequently, with 54 samples; excessive efficacy claims and insufficient risk warnings co-occurred in 47 samples; and excessive efficacy claims and unclear applicability boundaries co-occurred in 23 samples. Further analysis of specific combinations revealed that “unclear applicability boundaries + insufficient risk warnings” was the most common, with 35 samples (14.0%); “excessive efficacy claims + insufficient risk warnings” occurred in 29 samples (11.6%); and videos with exactly B3, C3, and D3, without E3, totalled 16 (6.4%). The main high-risk combination patterns are shown in Table 11.

**Table 11:** Main high-risk combination patterns.

Combination pattern	Sample count	Sample proportion
No grade 3 problems	114	45.60%
C3 + D3	35	14.00%
B3	32	12.80%
B3 + D3	29	11.60%
B3 + C3 + D3	16	6.40%
D3	9	3.60%
B3 + C3	5	2.00%
C3	5	2.00%

B, C, D, and E denote efficacy claims, applicability boundaries, risk warnings, and professional justification, respectively. The suffix 3 denotes a score of 3; + denotes co-occurrence within the same video. Rows are mutually exclusive exact combinations: dimensions not listed in a row do not score 3. Pairwise co-occurrence counts in the text also include higher-order combinations. The eight displayed patterns account for 245 videos (98.0%); other low-frequency patterns account for 5 videos (2.0%) and are not displayed. All percentages use 250 videos as the denominator.

## 5 Discussion

### 5.1 The tension between low-threshold following along and expressive boundaries

The coding results of this study show that the primary problem with low-threshold follow-along exercise-health short videos lies in the frequent failure to simultaneously communicate efficacy conditions, target populations, risk warnings, and justifications within the imitable expressive format. Among the 250 samples, 92 videos (36.8%) received a score of 3 on the risk warning dimension, 86 (34.4%) on the efficacy claims dimension, and 64 (25.6%) on the applicability boundaries dimension. This indicates that risk identification for follow-along content cannot focus solely on the movements themselves; it must also examine whether the video provides sufficient judgmental information for ordinary users.

Fitness short videos warrant separate discussion because they differ from general health science communication—the former points toward “what to do” and “how to do it”, rather than merely influencing what users “know”. Existing research has explained how fitness short videos translate viewing into imitative impulses and bodily practice through concepts such as “participability” and multimodal communication (7, 8, 12, 15). This “follow-along-ability” elevates the importance of boundary expression. Studies have found that the informational value, source credibility, and interactive experience of fitness short videos influence users’ browsing engagement and are associated with offline exercise participation willingness (9). When videos organize content with phrases like “follow me”, “do it daily”, “relieve pain”, or “slim down fast”, the expressions in titles, covers, voiceovers, and subtitles become important information sources for users deciding whether to follow along. The high proportions of scores 2 and 3 across dimensions B, C, and D in this sample are precisely the manifestation of such expressive information insufficiency at the content level.

Different content types exhibit different expressive problems. Pain relief/general rehabilitation expression videos have the highest proportions of score 3 on applicability boundaries and risk warnings (86.4 and 81.4%, respectively); fat loss and body shaping videos are most prominent on the efficacy claims dimension, reaching 86.0%. Pain relief content often uses low back pain, neck and shoulder pain, or knee pain as entry points; without specifying unsuitable populations, stopping conditions, and situations requiring medical consultation, users will find it difficult to judge suitability on their own. Fat loss and body shaping content, in contrast, tends to reinforce outcome expectations through expressions such as “rapid weight loss” and “localized body shaping”, with problems concentrated between outcome expression and training load explanation.

The efficacy claim problems in fat loss and body shaping content mainly manifest as videos directly binding goals such as “slimming” and “fat burning” to movements, without establishing whether these expressions changed users’ expectations of practice outcomes. The coding in this study can only indicate that such expressions are relatively common in the sample; it cannot be used to judge actual conversion effects or communication mechanisms. The lack of applicability boundaries and risk warnings in pain relief/general rehabilitation content is closer to a gray-area problem situated between fitness guidance and rehabilitation expression: without specifying unsuitable populations, stopping conditions, and further assessment needs, ordinary users find it more difficult to judge whether they are suitable to follow along. Although stretching, desk exercise, and Baduanjin/wellness exercise content generally has lower overall intensity, applicability boundaries remain widely incomplete, indicating that low intensity does not equate to being able to omit boundary specifications.

From the perspective of risk society theory (22–25), these findings are better understood as a forward shift in individualized risk judgment: in follow-along scenarios, users often need to rely on the video itself to judge whether it suits them, whether there is a risk of pain exacerbation, or whether movement loads are excessive. If the video does not specify target populations, risk warnings, and stopping conditions, the judgmental cues available to users decrease. In other words, the “incomplete expressive boundaries” revealed by this study’s coding mean that ordinary users are more likely to be pushed to the forefront in follow-along scenarios, independently completing risk judgments that should ideally be supported by more adequate information.

### 5.2 From information quality to follow-along expressive boundaries

The concept of “expressive boundary” used in this study functions not to judge whether video content is right or wrong, but to identify whether the video provides users with the key information needed for following along. This analytical orientation differs markedly from existing evaluation tools such as DISCERN and JAMA benchmarks—the latter take information reliability, evidentiary adequacy, and content comprehensibility as core indicators, with their judgment logic premised on whether information is “true.” However, the problem with follow-along exercise-health videos often lies not in whether the movement demonstration itself is “correct”, but in the video’s failure to specify the target populations, intensity boundaries, and stopping conditions for practice. For this reason, this study shifts the analytical focus from “whether information is true” to “whether information is complete”.

Existing research has established evaluative references for exercise-health short videos across multiple dimensions, including information reliability, content completeness, and educational suitability (10, 42, 46). However, these studies are mostly focused on specific disease topics such as scoliosis, lumbar disc herniation, and knee osteoarthritis, and their analytical frameworks are difficult to transplant directly to the low-threshold daily content scenarios of ordinary users following along. The coding data in this study show that only 2.0% of samples received a score of 3 on the professional justification dimension, but 75.6% received a score of 2—this distribution pattern indicates that samples rarely show obvious false authoritative packaging; more commonly, videos propose goals of health improvement, pain relief, or postural correction without specifying movement sources, applicable rationales, or professional justifications. In other words, the core problem is not “false statements” but “insufficient information.” Existing research has also shown that there is no simple correspondence between the quality level of health information on short-video platforms and communication effects; user interaction data cannot serve as a substitute indicator for information quality (54). In the professional justification dimension, a health claim without stated sources, identity, or movement rationale is typically scored as 2. A score of 3 is assigned only when unverifiable authoritative packaging—such as claims to medical, expert, authoritative, or secret-remedy status—is combined with strong health claims or therapeutic expressions, or when professional identity clearly mismatches therapeutic expressions. Account nicknames or vague identity cues do not by themselves constitute sufficient professional justification and do not automatically trigger a score of 3.

The “expressive boundary” framework constructed through the four dimensions of efficacy claims, applicability boundaries, risk warnings, and professional justification is designed precisely to identify this type of “partially correct but incomplete” information form. This framework does not replace the judgment of “truthfulness” by tools such as DISCERN; rather, it addresses a question that has not yet been fully covered: whether videos provide the informational conditions users need to judge their own suitability.

### 5.3 Collaborative governance of expressive boundaries

From a governance perspective, the results of this study suggest that low-threshold follow-along exercise-health content cannot be handled merely as “false information” or “dangerous actions.” The co-occurrence analysis in the sample shows that unclear applicability boundaries and insufficient risk warnings co-occur in 54 videos, and excessive efficacy claims and insufficient risk warnings co-occur in 47 videos, indicating that insufficient risk warnings are often the convergence point of multiple boundary problems. The problem with some videos is not exaggerated wording alone, but the failure to form a complete explanatory chain among effects, targets, risks, and justifications.

National policies have already set forth principled requirements for scientific fitness and risk prevention. The Outline of the “Healthy China 2030” Plan emphasizes exercise risk assessment for different populations and environments (55); the National Fitness Plan (2021–2025) calls for improving the level of scientific fitness guidance services (56); and the Guiding Opinions on Promoting High-Quality Development of Sports for Health further emphasizes improving the public fitness service system and accelerating the deep integration of national fitness and public health (2). These policies collectively point to the need for exercise-health guidance to distinguish target populations, specify exercise intensity, and indicate exercise risks—precisely what Dimensions C (applicability boundaries) and D (risk warnings) in this study assess. However, when implemented in short-video follow-along scenarios, these requirements need to be further translated into specific expressive requirements: “who is suitable, how much to do, when to stop, and on what basis”.

The task priorities of different actors should be differentiated. Creators need to supplement information according to different video types: fat loss and body shaping content should specify outcome conditions and training intensity; pain relief/general rehabilitation content should specify unsuitable populations, contraindicated situations, and stopping conditions; posture correction content should avoid reducing complex physical states to single movement outcomes; and although stretching and wellness exercise content has lower intensity, it still needs to specify applicability conditions. Platforms should identify high-frequency problems such as exaggerated effects, missing contraindications, and insufficient risk warnings, and should set up categorized prompts, risk warning templates, and high-risk expression review rules. Research on science communication has shown that professional identity, commercial relationships, and backstage information disclosure affect audience trust judgments, and the value of information disclosure lies in helping users understand information sources and judgment bases (48). Therefore, platform governance should not stop at displaying identity labels but should place its focus on ensuring information completeness. Professional institutions can provide guidance on movement descriptions, risk warnings, and scientific fitness expression. Regulatory authorities need to clarify the boundary lines between general exercise guidance, health improvement expression, disease-related expression, and commercial promotion.

Research on smoking cessation short videos has proposed a collaborative framework involving government, platforms, experts, and users, which can serve as an external reference for health short-video governance (57). What this study can offer is governance implications based on sample expression problems, rather than empirical evaluations of platform review processes or governance effects.

### 5.4 Research limitations

This study’s research subjects are limited to publicly visible low-threshold follow-along exercise-health short videos on the Douyin platform. The sample can reflect the expressive characteristics of public videos within the collection scope, but it cannot represent all platforms, all exercise-health content, or users’ actual follow-along behaviors. The sample screening and coding processes have been conducted to ensure link verifiability, content reviewability, and evidence location recordability as much as possible. However, short-video content is characterized by continuous updates, deletions, and changes in recommendation rankings. Future research could expand the platform scope and repeat collection at different time points to compare whether content expression changes.

This study adopts a diagnostic evaluative content analysis method, with the core task of identifying problems in efficacy claims, applicability boundaries, risk warnings, and professional justification in video expression, rather than testing whether the movements themselves are effective or tracking whether users are injured by following along. Therefore, the results of this study are suitable for explaining expression problems, type differences, and co-occurrence patterns of problems within the sample, but are not suitable for directly stating injury incidence rates, actual health consequences, or platform governance effects. If future research aims to answer whether users follow along, whether they are injured, how platforms review, and whether governance measures are effective, it will need to further integrate user interviews, platform rule enforcement materials, complaint handling data, or experimental research designs.

Type comparisons are also affected by context-specific scoring rules. Dimension E had only 5 score-3 samples, and this low-frequency distribution limits the identification of differences. This study used exact tests to handle sparse cells; future research could expand the sample and compare results under different scoring thresholds.

## 6 Conclusion

Following the analytical path of “identifying problems—comparing differences—analyzing co-occurrence—exploring governance”, this study examines boundary expression problems in low-threshold follow-along exercise-health short videos. Based on 250 Douyin platform samples and diagnostic analysis across the four dimensions of efficacy claims, applicability boundaries, risk warnings, and professional justification, the findings reveal: 92 samples (36.8%) with score 3 on risk warnings, 86 (34.4%) on efficacy claims, 64 (25.6%) on applicability boundaries, and 5 (2.0%) on professional justification. Prominent problems are concentrated in risk warnings, efficacy claims, and applicability boundaries; although severely problematic samples are few in the professional justification dimension, 75.6% of samples received score 2, indicating the problem manifests more as insufficient justification rather than false authoritative packaging.

In terms of overall distribution, dimensions B, C, and D are predominantly composed of scores 2 and 3, indicating that problems are not limited to a few extreme videos but more commonly manifest as incomplete information or unclear judgmental conditions. A total of 136 samples have at least one score-3 high-risk problem (54.4%), with 90 having two or more simultaneously (36.0%). This indicates that high-risk problems are not isolated but tend to concentrate in the same video in a superimposed manner; some videos simultaneously exhibit insufficient expression across effects, applicability conditions, and risk explanations. The core judgment of this study thus converges on “incomplete expressive boundaries”.

Different content types exhibited differentiated boundary expression problems. Fat-loss and body-shaping videos had a score-3 proportion of 86.0% on the efficacy-claims dimension; such content more often contained expressions that directly bound goals such as rapid fat loss and localized shaping to movements. This study cannot determine whether such expressions altered users’ actual expectations. Pain-relief and general rehabilitation videos had score-3 proportions of 86.4 and 81.4% on the applicability-boundaries and risk-warnings dimensions, respectively; content involving pain or physical discomfort more often required specification of target populations, contraindicated situations, and stopping conditions. The two problem types had different emphases, and no simple overall risk ranking could be established.

The co-occurrence results of high-risk problems further demonstrate that evaluation cannot solely focus on the presence of false information or dangerous actions. C3 and D3 co-occur in 54 videos, B3 and D3 in 47; the “unclear applicability boundaries + insufficient risk warnings” combination appears in 35 videos (14.0%), the “excessive efficacy claims + insufficient risk warnings” combination in 29 (11.6%), and 16 videos (6.4%) exhibit exactly B3, C3, and D3, without E3. This indicates that some videos, while emphasizing movement effects, fail to specify who is suitable to practice, to what extent, and when to stop.

Based on the above findings, governance should shift from simply judging whether content is false to refining evaluations of expressive completeness. Creators should specify target populations, contraindicated situations, and stopping conditions according to content type; platforms can improve information completeness through content identification and risk warning templates; professional institutions can provide movement justifications and risk warning guidelines for ordinary users; regulatory authorities need to clarify the expressive boundaries between exercise-health content and disease treatment or commercial promotion.

This study extends the evaluative scope of “expressive boundaries” from “whether content is true” to “whether information is sufficient to support user judgment”, addressing the limitation of existing health information quality research, which has mostly focused on reliability, transparency, and comprehensibility while paying limited attention to the completeness dimension in executable information scenarios. Through four operational dimensions, this concept is transformed into a codable, comparable observational tool, and based on sample data, reveals structural differences in problems across content types. In terms of research positioning, the value lies not in proving that low-threshold follow-along videos have caused user harm, but in revealing the information deficiency at the expressive level, providing empirical references for subsequent governance discussions and rule refinement.

## Data Availability

The raw data supporting the conclusions of this article will be made available by the authors, without undue reservation.
